# Imaginaries of omniscience: Automating intelligence in the US Department of Defense

**DOI:** 10.1177/03063127221104938

**Published:** 2022-06-23

**Authors:** Lucy Suchman

**Affiliations:** Lancaster University, Lancaster, UK

**Keywords:** militarism, data, closed world, military technologies, imaginaries

## Abstract

The current reanimation of artificial intelligence includes a resurgence of investment in automating military intelligence on the part of the US Department of Defense. A series of programs set forth a technopolitical imaginary of fully integrated, comprehensive and real-time ‘situational awareness’ across US theaters of operation. Locating this imaginary within the history of ‘closed world’ discourse, I offer a critical reading of dominant scholarship within military circles that sets out the military’s cybernetic model of situational awareness in the form of the widely referenced Observe, Orient, Decide, Act or OODA Loop. I argue that the loop’s promise of dynamic homeostasis is held in place by the enduring premise of objectivist knowledge, enabled through a war apparatus that treats the contingencies and ambiguities of relations on the ground as noise from which a stable and unambiguous signal can be extracted. In contrast, recent challenges to the closed-world imaginary, based on critical scholarship and investigative journalism, suggest that the aspiration to closure is an engine for the continued destructiveness of US interventions and the associated regeneration of enmity. To challenge these technopolitics of violence we need a radically different kind of situational awareness, one that recognizes the place of ignorance in perpetuating the project of militarism. Only that kind of awareness can inform the public debate required to re-envision a future place for the US in the world, founded in alternative investments in demilitarization and commitments to our collective security.

During the past two decades the commitment on the part of the US Department of Defense (DoD) to what Edwards (1996) has analyzed as a ‘closed world’ of containment and military dominance has taken on new life. The shift during the 1990s from a frame of superpower conflict to the so-called irregular warfare of counterinsurgency and counterterror operations aligned well with the building out of networked infrastructures. Yet the associated Revolution in Military Affairs has resulted less in the dissolution of the persistent ‘fog of war’ than in its intensification.^1^ Read as a lack of information integration, the intransigent disorders of warfighting underwrite ever expanding investments in what I will argue is a resilient fantasy of data-driven, comprehensive command and control. Building out systems of sensors, signal processing, data storage and transmission has proven more straightforward than the translation of data into what in military terms is named ‘actionable intelligence’. As the excess of data now threatens to destabilize the technopolitical imaginary^2^ of just-in-time information, artificial intelligence (AI) is advanced as the promissory solution to automating data analysis and reclosing the world.

In this article I trace how the figure of AI^3^ has been mobilized by an alliance of military technophiles and commercial entrepreneurs as a means of responding to what they characterize as the new demands of 21st century warfighting. The question that guides my analysis is how commitments to containment sustain themselves across changing circumstances of warfighting and associated infrastructures. Building upon theoretical resources afforded by scholarship in science and technology studies (STS), I begin by reviewing the frame of closed-world discourse. Edwards (1996) and Masco (2014) trace the progression of closed-world discourse as it underwrites associated investments in rationality and control through systems engineering, from the Cold War to the war on terror. I extend this line of analysis to what I read as the re-articulation of closed world discourse in the current revival of AI, including the promise to delineate and dominate the theater of operations through data. I suggest that what holds this promise in place is the enduring premise of objectivist knowledge enabled through a war fighting apparatus that treats the contingencies and ambiguities of relations on the ground as noise from which a stable and unambiguous signal can be extracted. That premise can only be challenged through recognition of the performativity of the military apparatus in regenerating the realities that it is trained to see, and through alternative knowledge-making practices based in critical analysis of received assumptions and careful investigation of the sites and consequences of military operations.

I begin with a review of the closed-world imaginary as elaborated by Edwards and Masco, to follow the through lines that connect Cold War military doctrine to contemporary operations in the name of counterterror.^4^ Within the closed-world imaginary, the task of military intelligence is to maintain a kind of dynamic homeostasis, nominalized in military discourse as ‘situational awareness’. In the second section of the article, I offer a critical reading of dominant scholarship within military circles that sets out the military’s cybernetic model of situational awareness in the form of the widely referenced Observe, Orient, Decide, Act or OODA Loop. Rendered as a cognitive system aiming for dominance through equilibrium, situational awareness sustains the premises of rationality necessary for the operations of command and control. I turn next to show how those premises provide the conditions of plausibility for current calls, most prominently by the National Security Commission on AI (NSCAI), for AI-enabled automation of military intelligence as a means of dynamically expanding and reconfiguring, and in that way re-containing, the theater of war. That call is taken up in the current project of Joint All-Domain Command and Control (JADC2), a vision of ‘sensor to shooter’ infrastructures enabled through Machine Learning (ML) that will render the battlespace transparent (to us) but also secure (from them).

With this context in mind, I turn in the article’s final section to recent challenges to the closed-world imaginary, based on critical scholarship and investigative journalism, including forensic analysis of the destruction wrought through military operations and other modes of research and reporting on-the-ground. These investigations provide evidence for the continued escape of conflict from the frames of rational action and control on which militarism depends, manifest in acts of extrajudicial assassination and the injury and death of those categorized as civilians. These accounts not only challenge the military’s attempt to make clean demarcations of enmity within complex relations of affinity and difference, but also offer an example of partial, specifically situated and highly illuminating knowledge-making practices outside of the military’s imaginaries of omniscience. They make evident that rather than perfect information, those imaginaries are founded on expedient and systemic ignorance.

In conclusion I argue that the aspiration to closure itself is the source of the continued destructiveness of US interventions and the associated regeneration of enmity. The commitment to close the world drives the technosolutionism offered most recently by AI. To challenge that we need a radically different conception of situational awareness, in the form of public debate and a re-envisioning of the future place of the US in the world, founded in comparable investments in creative diplomacy and a transition to demilitarization.

## Closed world, redux

Since the United States’ arguably forced entry into WWII, US foreign policy has become a mandate for global military supremacy (Wertheim, 2020). Reading this history, Edwards (1996) puts forward the metaphor of the ‘closed world’ or ‘dome of global technological oversight’ to describe an ideological stance that emerged in the context of the Truman doctrine of containment in 1946, was elaborated in the surveillance technologies and guided weapon systems of the Vietnam war during the 1960s and 70s, and culminated in the US-Soviet arms race of the 1980s (see also González, 2021). That technological imaginary inscribed its politics into the buildout of a computationally based military machine devoted to command and control:

Computers made the closed world work simultaneously as technology, as political system, and as ideological mirage. … Both the engineering and the politics of closed-world discourse centered around problems of human-machine integration: building weapons, systems, and strategies whose human and machine components could function as a seamless web, even on the global scales and in the vastly compressed time frames of superpower nuclear war. As symbol-manipulating logic machines, computers would automate or assist tasks of perception, reasoning, and control in integrated systems (Edwards, 1996: 1-2).

Edwards observes that while the Cold War nuclear stand-off strengthened the role of simulations, war games, and computer modeling as proxies for weapons that could not be used, the translation of the superpower conflict to the ground in Southeast Asia beginning in the 1960s afforded a different experimental site for closed-world thinking. Operation Igloo White, the project of instrumenting the Ho Chi Minh Trail with sensors to disable it as a North Vietnamese transport road, embodied a fantasy of total surveillance and complete control over the battlefield from the safety of a distant, high-tech command center (the Infiltration Surveillance Center or ISC in Thailand), along with wired-in, computer-controlled aircraft cockpits (see also Chaar López, in press; Elish, 2017). Together these and other developments resulted in an extreme intensification of efforts toward (and claims for) centralized, remotely controlled operations based on advanced computing and communications. They disclosed as well ‘a wide gap between an official discourse of success and the pessimistic assessments of independent observers, including American soldiers on the ground’ (Edwards, 1996: 6), a difference to which I return below.

The second attack on the US ‘homeland’, on September 11, 2001, provided a platform for the launch of the current – and ongoing – program of counterterrorism operations around the world. Characterized by Chamayou (2014: 53) as more hunting ground than battlefield, the US military now favors ‘pop up’ bases over long-term occupations, and special forces, private contractors, and partner training over ‘boots on the ground’. Programs like the US led operation ‘Inherent Resolve’ against the Islamic State, inaugurated in 2014 and continuing as of this writing, rely upon air strikes and local partnerships across the Middle East and northern Africa, still treated in many respects as spaces of (colonial) trial and experimentation. All of this, as Demmers and Gould (2021) observe, enables greater obfuscation and less accountability for military actors. Highly secretive, these operations rest their legitimacy on claims for the use of ‘precision’ technology. However, as Chamayou (2014: 143) has observed, ‘The fact that your weapon enables you to destroy precisely whomever you wish does not mean that you are more capable of making out who is and who is not a legitimate target’. The language of precision in this context conflates the accuracy with which a weapon strikes a designated target with the vagaries through which US forces identify who and what warrants attack (Suchman, 2020).

In *The Theater of Operations*, Masco (2014) traces these lines of continuity across shifts in military strategy. We see a repetition, Masco observes, from the launch of the national security state in 1947 after the Pearl Harbor attack (with the passage of the National Security Act that created the DoD, the National Security Council, and the CIA) to the national security state of the early 2000s (with the Authorization for the Use of Military Force and the initiation of Special Forces). Both moments involve what Masco sums as the ‘designation of new insecurities, new institutions to fight them, a public mobilization campaign grounded in fear’, along with the declaration of a new mode of life rather than a specific period of conflict (p. 5). Insecurity is the condition of possibility for militarism on this analysis, kept alive by an imagined world of perfect preparedness against an open horizon of threat. The result is a privileging of military investment that arguably reproduces the very conditions that it purports to resolve:

By allocating conceptual, material, and affective resources to ward off imagined but potentially catastrophic terrorist futures, the counterterror state also creates the conditions for those catastrophic futures to emerge. It does so by generating new arms races; increasing international blowback from war, covert actions, and drone strikes; and by not responding to existing suffering at home and abroad with the same urgency (Masco, 2014: 13).

With these analyses as background, I turn to what I take to be a core premise that connects successive revolutions in military affairs, however they are configured. Despite the manifest performativity of these discourses of insecurity, the closed world and its theaters of operation rest upon an objectivist onto-epistemology that takes as self-evident the independent existence of a world ‘out there’ (Law, 2004) to which military action is a necessary response. Powered by the trope of ‘situational awareness’ as the objective function of military intelligence, the dome of global technological oversight described by Edwards is being refashioned, as I elaborate below, in the latest call for investments in AI. In the next section I trace the behaviorist and cognitivist genealogies that inform models of knowing and acting within military imaginaries and the associated discourse of situational awareness, as these continue to underwrite the military’s commitment to a model of itself as a controllable machine.

## Behaviorist/cognitivist genealogies of situational awareness

A core precept of modern military doctrine is the requirement for situational awareness, defined as ‘the perception of environmental elements and events with respect to time or space, the comprehension of their meaning, and the projection of their future status’ (Endsley, 1995: 36). The logics that inform contemporary strategies of AI-enabled situational awareness remain firmly rooted in cybernetic meta-theory and its influence on contemporaneous developments in the psychological sciences in the mid-20th century (see Bowker, 1993). With stimulus and response as its core concepts, the behaviorist paradigm took as its evidentiary base experimental setups where the stimulus was characterized as some aspect of an environment understood as external to the actant, and the response as any observable movement taken to be effected by exposure to the stimulus. Following the cybernetic model, a ‘torpedo with a target-seeking mechanism’ could be said to exhibit ‘intrinsic purposeful behavior’ (Rosenblueth et al., 1943: 2). As Edwards (1996: 184) notes, ‘What is involved in the practice of definition and “selection” of goals, something torpedoes and guided missiles do not do for themselves, was not discussed’. With Shannon’s (1949) formalization of the terms ‘information’ and ‘communication’ so that meaning became irrelevant to either, the foundations for a computational imaginary of cognition and interaction were in place.

**Figure 1.**
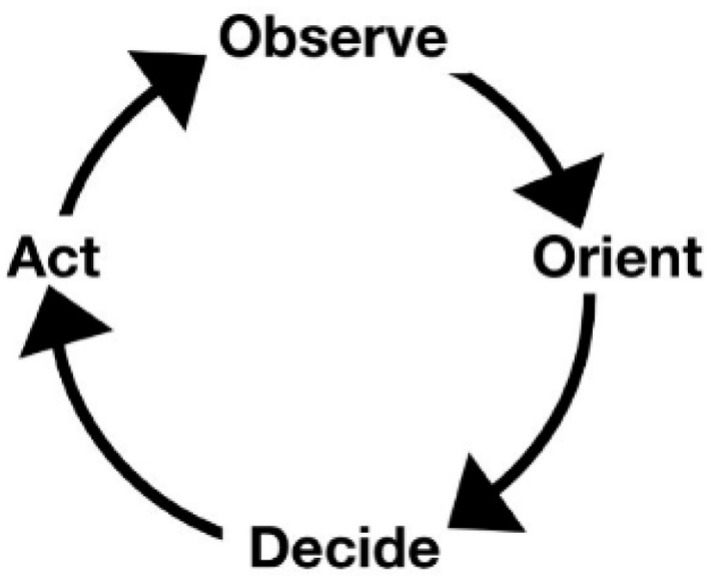
The OODA loop depicted as a simple sequential process. Reproduced in Richards (2020: 144).

Embraced by military technophiles and strategists, information processing psychology provided a comprehensive model of rational action fit for modern war fighting. Incorporated into the cybernetic loop, the human became the ‘man in the middle’ of the military machine. In contemporary military discourse, this is the ‘human in/on the loop’ of complex cybernetic weapon systems. The figure of the ‘loop’ is institutionalized as the canonical cycle Observe, Orient, Decide and Act (OODA) first set out by US Air Force Colonel John R Boyd in a series of unpublished and unclassified DOD briefing papers (Boyd, 1996) (see Figure 1). As a discursive artifact with continuing agency in the military imaginary,^5^ the OODA loop warrants closer analysis.

While Norbert Wiener’s cybernetic models were developed in the service of guidance for anti-aircraft weaponry, Boyd’s grew out of his experience as a fighter pilot and instructor. In a recent reading of Boyd, Richards (2020) emphasizes that despite the common depiction Boyd never actually figured his conception of the loop as a sequential or circular course of action. Boyd treated sensing, Richards emphasizes, as the precondition for observation. At the same time, he saw the loop itself as a precondition for action: ‘Without OODA loops, we can neither sense, hence observe, [and] thereby collect a variety of information … nor decide as well as implement actions’ (Boyd, 1996: 1). But the critical point according to Richards is that the original loop over-represented the place of decision in military action:

most of the time our actions should flow from orientation directly and implicitly, that is, without explicit (e.g., written or detailed verbal) commands or instructions. Such ‘implicit guidance and control’ is difficult to model with the loop of Figure 1, which contains an explicit ‘decision’ step that every cycle must pass through (2020: 146).

**Figure 2.**
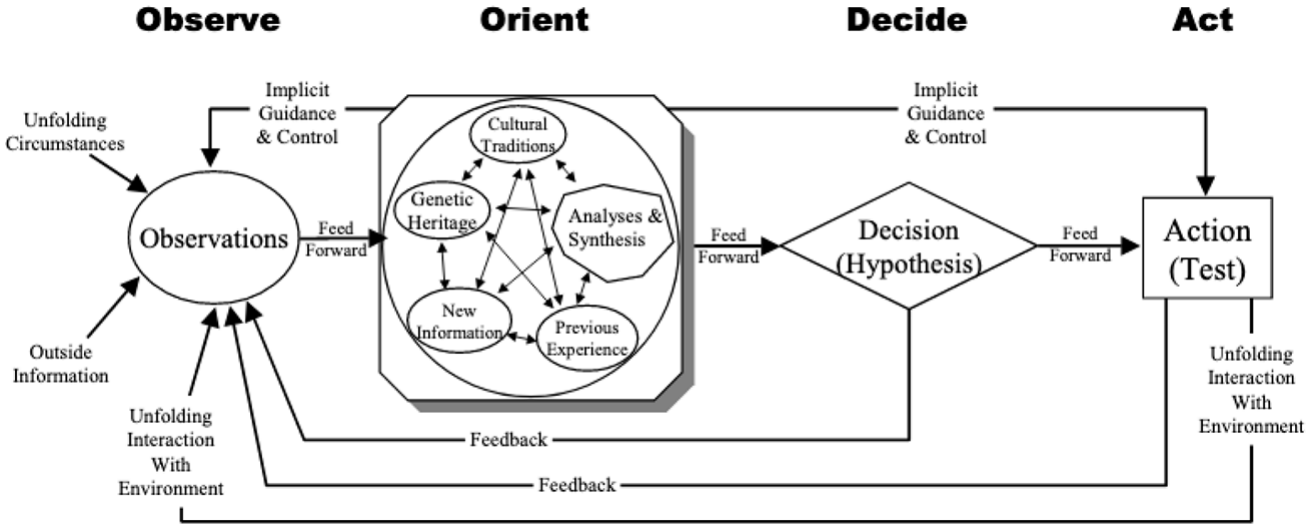
The modified OODA loop. Reproduced in Richards (2020: 146) after Boyd (1996), ‘The essence of winning and losing’.

In a more cybernetic mode, Boyd himself diagrammed the OODA loop as shown in Figure 2. Richards’ offers this cognitivist reading of the diagram:

The entire Orientation bubble, including the blocks and all the interactions between them, represents our mental models of reality that are making predictions about the effects of our actions. The ‘Observation’ bubble includes all the ways we bring in information both from the external world as well as about our own bodies and minds. (2020: 146)

This elaborated diagram makes clear that for Boyd the complexity is located at the boundaries of observation (with its externalized inputs including ‘unfolding circumstances’ and ‘outside information’,) and orientation (with its internalized components comprising ‘cultural traditions’, ‘genetic heritage’, ‘new information’, ‘previous experience’, and ‘analyses and syntheses’). The cybernetic circuit occurs when ‘Implicit Guidance and Control’, a kind of autonomic function, feeds back into observation along with ‘Unfolding Interaction with Environment’, that is, the effects of actions taken.

**Figure 3.**
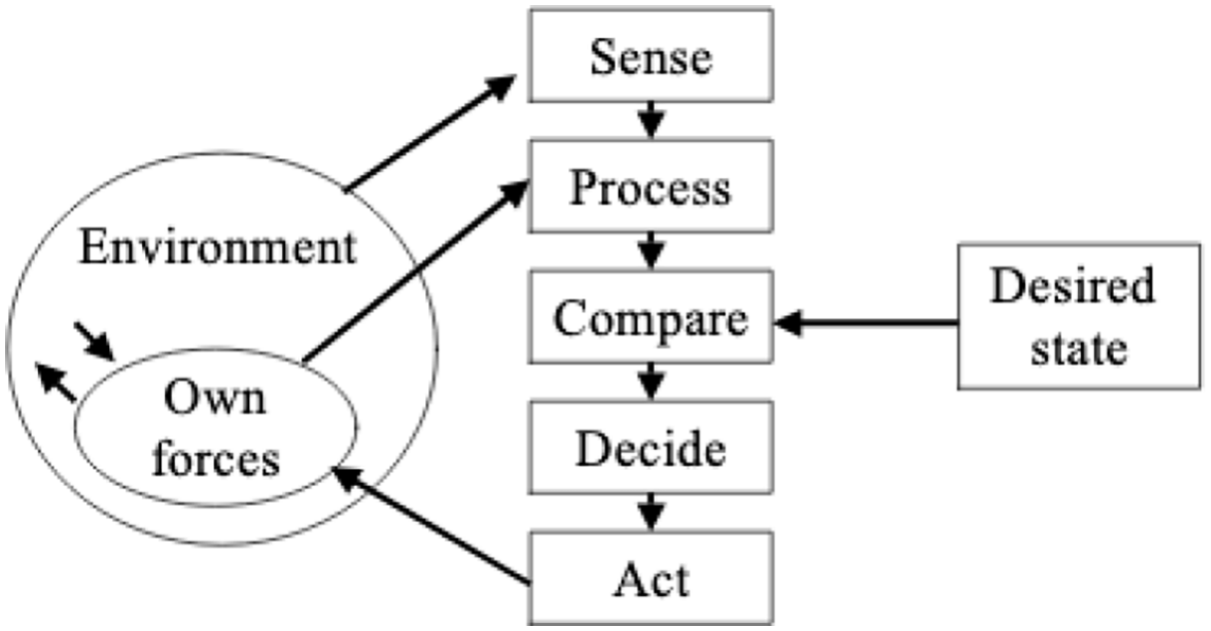
Lawson’s model. Reproduced in Brehmer (2005: 6).

Within the context of war fighting, effective operations under the OODA model require that ‘our’ side have a shared Orientation, what Boyd called an integrated, consistent ‘overall mind time-space scheme’ or a ‘common outlook [that] represents a unifying theme that can be used to simultaneously encourage subordinate initiative yet realize superior intent’ (Boyd, 1986: 74). At its imagined ideal, this shared mental model obviates the need for explicit command and control, as the force operates as a single body. Brehmer (2005: 2) calls for an ‘amalgamation’ between the OODA loop and cybernetic models of command and control, in what he names the ‘Dynamic OODA-loop, or DOODA-loop for short’. He points out that with his Figure 2, ‘Boyd achieved generalization of the OODA loop by elaborating the Orient stage from representing a physical orientation to representing a mental orientation and by introducing a number of feedback loops, thus actually placing the OODA-loop in the cybernetic camp’ (Brehmer, 2005: 3–4). Notably, this characterization also maintains the positioning of the military conception of situational awareness firmly within the rationalist/cognitivist camp. At the same time, Brehmer argues that cybernetics expands the loop to include ‘a representation of the environment that is affected by the decision maker’s actions’ (p. 5). He cites Lawson (1981), who proposes a model of cybernetic control that explicitly represents the ‘Environment’, while maintaining the loop as a closed world (see Figure 3).

Since the 1990s, the OODA frame has continued to serve as a normative model of situation awareness and associated training pedagogy, promoted most successfully by Micah Endsley, former Chief Scientist of the United States Air Force. Like Boyd, Endsley’s frame of reference is the closed world of fighter pilots and crews. In a 1997 report to the US Air Force titled ‘Situation Awareness, Information Dominance & Information Warfare’, Endsley and colleagues propose that Situation Awareness (SA) in their model can be seen as a more detailed account of the Observe and Orient stages of the OODA loop, which they represent as the simple 4-part cycle shown in Figure 1 above. Contra Richards (2020), they attribute that figure to Boyd and state that he equates it to the command and control loop (1997: 11–12), where it needs to operate across a hierarchy of levels. Within this framework, actors operate as ‘individual information processors in a collective manner’ (p. 13).

Acknowledging the network-centric warfighting of the latest revolution in military affairs (Owens et al., 2000), Endsley and her colleagues reassert the relevance of situation awareness, ‘loosely defined as knowing what is going on’, to the goal of information dominance:

It is expected that US forces will need to operate at considerable distances from home and existing bases as dictated by the actions of unpredictable hostile forces in various areas of the world. This necessitates ‘global awareness’ – information of suitable resolution and short update rates from any location on the globe. (Endsley and Jones, 1997: 4)^6^

They note that additional characteristics of emerging formations are dispersed and networked units, and the increased tempo of operations, which they define as ‘the time period required to assess a situation and plan and carry out a military action’ (Endsley and Jones, 1997: 6). This then leads logically to a need for greater ‘autonomy’ and automation:

Dealing with the huge volume of data created by the distributed network of sensors will also be greatly facilitated by providing a direct ‘sensor-to shooter’ link. A concept such as ‘Brilliant Pebbles’, in which weapons (e.g., missiles or mines) are equipped with sensors, microchips, and detonation power, provides autonomous or semi-autonomous units with the ability to act on their own to predefined classes of threats or situations. (Endsley and Jones, 1997: 7)^7^

At the same time, Endsley et al. anticipate the current crisis in data processing and analysis and the turn to pattern recognition as a solution. What they characterize as a naturalistic model of decision making ‘specifies a process in which the decision maker’s perceived situation is categorized based on recognized classes of situations for which known courses of action apply … a process of situation recognition and pattern matching to memory structures in order to make rapid decisions’ (Endsley and Jones, 1997: 14–15). In language reminiscent of 1980s expert systems, actors are understood to be drawing upon ‘situation prototypes’ and a repertoire of ‘scripted actions’ (Endsley and Jones, 1997: 15). Thanks to the availability of these mental models, and anticipating the turn to data analytics, they posit that SA can become a process of efficient human pattern matching:

A major advantage is that the current situation does not need to be exactly like one encountered before due to the use of categorization mapping (a best fit between the characteristics of the situation and the characteristics of known categories or prototypes). Furthermore, this entire process can be almost instantaneous due to the superior abilities of human pattern matching mechanisms. (Endsley and Jones, 1997: 24)

Like the environment, information in Endsley’s model is taken to be extraneous and objectively independent of the actor(s), more and less accurately sensed and comprehended. With the advent of machine sensing and computational infrastructures for data storage at scale, the stage is set for a crisis in ‘human pattern matching’ for which the only solution is further computation. The task of addressing this lacuna falls to a public-private alliance of military technophiles and commercial, more specifically Silicon Valley, advisors.

## The long pitch: From the DIB to the NSCAI

In April of 2016 then Secretary of Defense Ashton Carter created the Defense Innovation Advisory Board (DIB), as an independent federal advisory committee charged with ‘catalyzing innovation across the Department [of Defense]’ (Defense Innovation Board, 2016b). The sense of independence here is a subtle one, until we understand that this means that the Board’s members are not currently employed directly by the DoD. The Board is positioned as helping to bring Silicon-Valley ‘best practices’ to the DoD, in association with the Defense Innovation Unit (DIU/X) based in Mountain View, California, modeled as a venture capital firm dedicated to funding ‘start up’ military R&D projects. The Board’s initial Chair is Eric Schmidt, former CEO and Executive Chairman of Google and then of Google’s parent company, Alphabet. The Board is managed by Executive Director Joshua Marcuse, former advisor to the Pentagon in the Office of the Under Secretary of Defense for Policy.^8^ DIB members include executives of Google, Facebook, and Microsoft, as well as senior management at elite technical universities who have long partnered with the DoD. The DIB Charter states that ‘All members of the Board are appointed to provide advice on the basis of their best judgment without representing any particular point of view and in a manner that is free from conflict of interest’ (Defense Innovation Board, 2016a). The Board’s work is set up as a parallel process including public meetings and panels with AI experts run by the DIB, and regular DIB consultation with an internal Pentagon working group.^9^ Recommendations are to be delivered to the Secretary of Defense ‘about the ways that AI should or should not be injected into weapons programs’ (Fryer-Biggs, 2018).

One year later, in April of 2017, the DoD announced plans for its flagship AI project, the Algorithmic Warfare Cross-Functional Team (code-named Project Maven) under the leadership of Lt General ‘Jack’ Shanahan. The memo that establishes Project Maven was signed by then Deputy Secretary of Defense Robert Work. The plan for Maven includes an initial project focused on the task of labeling ‘objects’ (which it soon became clear includes vehicles, buildings, and also persons) in the video generated by US drone surveillance operations, as a first step toward establishing the computational infrastructures needed to automate object detection and classification and to generate, in the words of Robert Work, ‘actionable intelligence and insights at speed’ (Deputy Secretary of Defense, 2017).

On June 27, 2018, a further memo from then Deputy Secretary of Defense, Patrick M Shanahan, established the Joint Artificial Intelligence Center (JAIC), to be led by former Project Maven head Lt. General ‘Jack’ Shanahan.^10^ Citing the 2018 National Defense Strategy, the memo charges the JAIC ‘with the overarching goal of accelerating the delivery of AI-enabled capabilities, scaling the Department-wide impact of AI, and synchronizing DOD AI activities to expand Joint Forces advantage’. The memo urges ‘all offices and personnel to provide all reasonable support necessary to make rapid enterprise-wide AI adoption a reality’ (Department of Defense, 2018).

As part of the John S McCain National Defense Authorization Act for Fiscal Year 2019 (NDAA), the US Congress created the National Security Commission on Artificial Intelligence (NSCAI). Eric Schmidt (recently retired from the DIB) was appointed Chair of the Commission and former Deputy Secretary of Defense Robert Work was appointed Co-Chair. As in the case of the DIB, members of the Commission comprised current and former CEOs and other senior managers of (big) tech companies (Amazon, Google, In-Q-Tel, Microsoft, Oracle), current and former members of the Defense and Intelligence agencies, and senior members of universities with extensive DoD funding (Caltech, CMU). Nominated to serve on the grounds that they have the relevant expertise, all arguably have vested interests in increased funding for AI research and development. Nonetheless, Chair Schmidt stated that in the commissioners ‘We ended up with a representative team of America’ (NSCAI, 2021a, my transcript).

In a public plenary held online on January 25, 2021, the NSCAI convened to discuss its final report and recommendations, prior to their formal adoption and delivery to Congress and the President.^11^ There was even more urgency to the Commission’s work at that point than when they started, Chair Eric Schmidt stated, as:

scientific advantages leveraging AI continue to accelerate. … For the first time that I can remember, the commercial sector is actually better equipped than the government in leading the technology edge in this area. We must bring that technology into the military and the intelligence agencies in the right way (NSCAI, 2021a, my transcript).

Schmidt’s statement sets out the premises of the Commission’s work: that the field of AI is a technoscience advancing beyond the pace of its practical application (and by implication that the latter needs to be accelerated in order to catch up), that military and intelligence operations are the priority (and by implication also appropriate) domains of application, that the commercial sector is best prepared to lead the effort and to guide correct implementation, and that the latter means maintaining the US moral high ground in adherence to democratic values and anti-authoritarian policies and practices.

In a plenary on February 17, 2021, the commissioners presented a preview of their final report. A slide summarizing Part 1of the report includes the propositions that ‘We can expect the large scale proliferation of AI-enabled capabilities’, ‘AI-enabled capabilities will be the tools of first resort in a new era of conflict’, ‘AI will transform all aspects of military affairs’, and ‘AI will revolutionize the practice of intelligence’ (NSCAI, 2021b). More faith-based than demonstrable, these statements belie the vagaries of just what AI is (e.g. Lanier and Weyl, 2020). While technologists understand AI as a convenient (and highly salable) shorthand for a suite of statistically based techniques and technologies for automating data analysis, the term as used throughout the report implies something singular and *sui generis*. Any demonstrated risks, limits, or vulnerabilities of existing technologies are treated by the Commission as grounds for further investment: Along with the premise of an unavoidable arms race between the US and China, this proposition takes any question of decisions not to pursue the development of AI technologies off the table (see Suchman, 2021).

While promoting AI’s incorporation into military systems, the Commission warns that ‘AI will compress decision time frames from minutes to seconds, expand the scale of attacks, and demand responses that will tax the limits of human cognition’ (NSCAI, 2021c: 25). The solution to problems generated by weapon system automation, it follows, must be increasingly autonomous weapon systems, based on the ‘AI promise – that a machine can perceive, decide, and act more quickly, in a more complex environment, with more accuracy than a human’ (NSCAI, 2021c: 24). Despite the lack of evidence to substantiate this promise, and the continuing international debate over the legality and morality of autonomous weapon systems, the Commission concludes that the US must pursue their development (Fryer-Biggs, 2021).

In the February plenary, NSCAI co-chair Robert Work sets out his summary of Chapter 3 of the Commission’s final report, titled ‘AI and warfare’. This chapter, he explains, reflects ‘our strong sentiment on how DoD and our warfighters have to position themselves to harness the incredible power and promise of AI-enabled technology, both on and off the battlefield, from the back office to the fox hole’ (my transcript). Work then offers this demonstration of moral reasoning:

The biggest contributor to inadvertent engagements is target misidentification. The Center for Naval Analysis did a study of I think it was almost a thousand engagements in Afghanistan, and 50% of the causes, 50% of hitting a non-combatant or a restricted entity, was caused by target misidentification. And in every case, they were done by humans. Humans make mistakes all the time in battle. And the hypothesis is, to be proven, that artificial intelligence will improve target identification, which should improve, and reduce the number of collateral damages, reduce the number of fratricides. So, it is a moral imperative to at least pursue this hypothesis. (NSCAI, 2021b, my transcript)

In a twist of syllogistic (non)argumentation, Work points out that as International Humanitarian Law (IHL) states that indiscriminate weapon systems are not legal, autonomous systems will not be in violation of IHL because they won’t be designed to be indiscriminate. Admitting the need to test automated targeting systems and understand their limitations, Work asserts that ‘there’s a lot of reason to believe that artificial intelligence will improve and decrease inadvertent engagements’.^12^ However unproven this proposition might be, Work implies, it would be a moral failure not to pursue it.

On March 1, 2021, with much media coverage, the NSCAI delivered its 750-page ‘Final report and recommendations’ to Congress (NSCAI, 2021c). The Commission’s pitch to Congress is that greatly increased investment is needed before AI’s promise can be realized. This in turn requires the development of ‘imaginative warfighting concepts to inform the development of AI-enabled capabilities’ as well as the definition of a joint warfighting network architecture by the end of 2021.

## Joint all domain command and control (JADC2)

The primary investment in a fully integrated command and control system at the time of this writing is named Joint All Domain Command and Control (JADC2). As summarized in the DoD-oriented publication *Defense One*: ‘In this vision of future warfare, everything on the battlefield is digitally linked, allowing artificially intelligent decision aides to help commanders find and hit targets’ (Tucker, 2021a). According to Vice Admiral Jeffrey Trussler, JADC2 is necessary because to achieve ‘that speed of decision … we have to link our systems, sensors, weapons, platforms together like we never have’ (Wolman, 2021). Trussler envisions future tools that ‘understand a specific target’ as well as all available weapon systems, to help a commander choose the best weapon system for the circumstances. Secretary of the Air Force Charles Pope promises that ‘As envisioned, JADC2 will allow US forces from all services – as well as allies and partners – to sense, make sense and act upon a vast array of data and information … fusing and analyzing the data with the help of machine learning and artificial intelligence and providing warfighters with preferred options at speeds not seen before’ (Pope, 2021).

Still in the process of conceptualization, the plans for JADC2 display a certain strategic vagueness. Speaking in April 2021, in anticipation of the delayed release of the JADC2 strategy, Vice Admiral Trussler admits that ‘That North Star is still out here. We’re still working to define it.’ As a consequence, he anticipates ongoing uncertainty:

If released, [the strategy] may actually raise more questions than answers. Because people are wanting to know what is the technical detail? What is the thing that is going to be delivered? It’s not going to talk about that. It’s going to talk about the strategy of how the services are going to need to approach this, how we’re going to need to collaborate. (Wolman, 2021)

The focus on collaboration indexes longstanding competition among the various services in relation to funding and to the procurement of new weapon systems. It is in that context that General Mark Milley, chair of the Joint Chiefs of Staff, has called for baseline standards for the multiply sourced systems that are anticipated, ‘to ensure that the data that comes off the vehicles, weapons, and equipment they produce can be shared across the military’ (Tucker, 2021a). That sharing, proponents acknowledge, is complicated not only by multiple vendors and lack of standards but also by the longstanding failures of cooperation among the services. At the same time, these acknowledgments are quickly followed by assurances that AI, supported by ‘an enterprise cloud solution’, is key to making the JADC2 vision real. As reported by Patrick Tucker in *Defense One*: ‘Artificial intelligence is seen as key to realizing the JADC2 vision, and moving to an enterprise cloud solution is key to running machine learning programs on large, streaming datasets across the Defense Department’ (Tucker, 2021a).^13^

After much contestation among competing vendors, in October 2019 the DoD awarded the Joint Enterprise Defense Infrastructure (JEDI) cloud computing contract, projected to reach $10 billion, to Microsoft. However subsequent court decisions in response to lawsuits from Amazon charging political interference (Jasper, 2021) have interrupted progress on the contract, forcing interim data storage infrastructure development back into the various services. The latter have also continued to run their own ‘experiments’. These include the Army’s Project Convergence, ‘aimed at rapidly accelerating the Army’s ability to find and take out targets by connecting people, vehicles and weapons through a massive, interconnected sensing and shooting kill web’ (Tucker, 2021b), the US Northern Command’s Global Information Dominance Experiment, and ongoing exercises coordinated across the Air Force, Army and Navy within the Special Operations Command. These exercises extend ongoing efforts at data integration between increasingly software-laden bodies, vehicles, and command locations, while continuing to be haunted by the demands and resistances of legacy systems.

While the details of these experiments are not public, we do know that as exercises they operate within a bounded environment comprising pre-designated targets. It is here that we find a crucial difference between sites of experimentation or trial, and sites of actual combat. Particularly problematic for weapon systems based on machine learning is the question of the sources for training data, and their associated translatability and transportability. The problem is evident in questions submitted by prospective vendors regarding the Joint Artificial Intelligence Center’s ‘Data Readiness for AI Development’ Request for Proposals in April 2021, particularly those asking for further specification of data requirements. Written questions referring to specific clauses in the Request include: ‘This section mentions “data” several times, but doesn’t provide detailed specifications. What kind of data (what format, what structure, etc.)? How large is the data? How fast does the data need to be moved? What sources will the pipeline be consuming from (APIs, databases, file systems, etc.)?’ Responses take the form of ‘Data labelling should be interpreted as the labelling or annotation of data sets for Artificial Intelligence training purposes’, or ‘This level of requirement detail is not available at the Basic Ordering Agreement (BOA) level’ (Joint Artificial Intelligence Center, 2021).

The problems sighted here are not simply a matter of early stages of system development. Caduff’s (2015) account of the early warning system developed to monitor emergent pandemics might just as well apply to the military imaginary of all-domain command and control:

the vision of a ‘global surveillance network’ and ‘early warning system’ described by experts in reports is often more virtual than real. The network is dispersed, the ties are thin, the surveillance erratic, and the meaning of the … information uncertain. The network is fragmented and fraught with inconsistencies. It is pictured as a seamless system of surveillance, but this is primarily the network’s own mythology of coherence, logic, and rationality. (p. 194).

Writing about the intersecting interests of security professionals and vendors of cloud storage solutions, Taylor (2021) follows the self-sustaining logics of ‘future-proofing’ as the mandate for the securitization of data. Taylor describes how, since the late 1990s, what remains of Cold War bunkers housing command-and-control centers around the world have been progressively repurposed as digital data storage facilities for cloud computing companies. By making the future of security reliant on data, he argues, ‘Big Data’ in turn generates the problem of its own securitization, for which cloud storage providers promise solutions. This argument can be expanded to the affiliated imaginary of AI-enabled military intelligence, in its reliance on an infrastructure of data services that are at once integrated, distributed, continually updated, and vulnerable to failure and to sabotage. As Taylor shows in the case of the bunkered data center, threatening futures are conjured within and through the regimes of securitization posited as a necessary response:

… Tsing has used the term ‘conjuring’ to capture the imaginative work that investment companies engage in to entice speculators. ‘In speculative enterprises’, Tsing observes, ‘profit must be imagined before it can be extracted; the possibility of economic performance must be conjured like a spirit to draw an audience of potential investors’ (2001: 159). If financial speculation is based on conjuring utopian futures of profit, the selling of bunkered cloud storage is based on conjuring the spectre of disaster. (Taylor, 2021: 85)

In the case of the long pitch of the Silicon Valley-military industrial complex and its embrace by promoters of the JADC2, utopian futures of profit and a conjured specter of disaster are conjoined. The disaster anticipated redoubles itself, as the promised solution to an insufficiency of data becomes a new site of vulnerability. The conjuring of a perfectly joined up military sensorium based in the cloud hides a more troubling lacuna than securitization, however, in its almost complete failure to address the onto-epistemologies of data’s generation.

## Prefigurations of the military sensorium and the limits of closure

In the NSCAI Plenary of Jan 25, 2021, Co-Chair Robert Work promises optimization of the operations ‘Prepare, Sense and Understand, Decide, Act’, capacities that he links to the canonical OODA loop theory’s Observe, Orient, Decide and Act (NSCAI, 2021a). Following Richard’s argument cited above, Work observes that the loop as envisioned by Boyd is not a closed, one-way cycle where Decide is most important, albeit that much discussion on AI-enabled systems goes toward decision-making tools. For Boyd, he explains, Orientation is the crucial step, informing the commander’s interpretations of the observations that are coming in, which then shape the decision, which in turn shapes the action. The objective, it follows, needs to be not to drive decisions but ‘to actually make sure that the commander’s perceived reality equals actual reality’. It is here that the problem arises for which, he posits, AI can offer solutions:

It is becoming more and more difficult to understand what actual reality is. And this in my view is where the AI tools can really help. They’re not gonna be right all the time, but I guarantee you that they’re going to be right more often than humans who are trying to understand an avalanche and tsunami of data. (NSCAI, 2021a, my transcript).

Work’s aspiration to ‘actionable intelligence at speed’ (Deputy Secretary of Defense, 2017) presumes transparent mediations and accountable translations from signal to data to information. In dominant military discourses this chain of reference (Latour, 1999) is considered problematic only in terms of its efficiency, rather than as a matter of its onto-epistemic premises. It is here that a growing body of investigative journalism and critical scholarship becomes relevant, posing fundamental questions for the project of automatic data analysis in the service of real-time feeds from ‘sensor to shooter’.

Johns (2016) identifies what she names the list-plus-algorithm as ‘an embedded juridical shortcut’ through which military actors propose to achieve the translation of signals, images, or textual traces for purposes of targeting. However, as she observes, ‘Surrounding the list-plus-algorithm in legal and policy settings, one may discern widening eddies of doubt amid rising oceans of data’ (p. 11). Johns questions calls for algorithmic ‘transparency’ as a solution to that doubt, insofar as transparency rests on the premise that if one can somehow get behind or beneath the list-plus-algorithm, further information will yield intelligibility. Instead, she argues, ‘behind listed data and encoded instructions for analysis one tends to encounter more data and data analysis practices, often stubbornly irreducible to one other’ (p. 16).

At the same time, productive forensics of the list-plus-algorithm are underway. Perugini and Gordon (2017) consider the generative effects of military investment in a machinery of situational awareness based on the subjection of big data to algorithmic analysis|:

Following Foucault’s (1980) notion of dispositif, we understand these new technologies and epistemic operations as part of an *apparatus of distinctio*n, which denotes an array of interrelated forces that frame, shape, produce, construe and thus give meaning to the relationship among the actors within the battlespace. (p. 8, my emphasis)

While the Latin roots of the word ‘discrimination’ refer to the general act of separating or making a distinction, since the late 19th century that term has acquired political significance in the context of ideologies associated with the prejudicial treatment of persons based on their racialized categorization (Apprich, 2019: ix–x). At the same time the term retains its original meaning in common usage. In fields like computer science ‘pattern discrimination’ is used as a technical term to describe algorithmic processes designed to sort input data to match a model of which differences are stipulated to matter. While adopting the term’s innocent etymology, the discriminations on which algorithmic pattern matching is built in many cases carry their injurious histories with them.^14^

In developing the guiding trope of ‘surrogate humanity’, Atanasoski and Vora (2019) tether the patterns of contemporary automation to much longer histories of subordination. Across the domains of ‘dull, dirty, and dangerous’ labor, from domestic servitude to fighting in wars, ‘Engineering imaginaries, even as they claim revolutionary status … tend to be limited by prior racial and gendered imaginaries of what kinds of tasks separate the human from the less-than or not-quite human other’ (p. 4). The freedom of the liberal subject, they argue, is only possible through the unfreedom of the surrogate, the latter being a racialized and gendered figure defining the limits of human consciousness and autonomy, whether in the body of the enslaved person or the machine. On this analysis, the imaginaries of the Joint All Domain Warfighting network can be understood as a continuation of an imperial impulse, within which ‘racial patterns of violence are continually reiterated as regulatory fields delimiting the fully human who must be protected’ (Atanasoski and Vora, 2019: 155). These patterns are what Wilke (2017: 2, original emphasis) characterizes as ‘*sociocultural prisms of visibility* in which technologies play an important enabling and legitimizing role’, a narrowing of vision that brings certain limited objects or events into sharp focus, while systematically placing more complex and unwieldy realities outside the frame.

In the case of so-called irregular warfare, what signals ‘participation in hostilities’ has shifted from an actor’s clothing and other emblematic designations to ‘what the actor does and the kind of relations s/he has with other human beings, the surrounding environment, and what International Humanitarian Law (IHL) defines as legitimate military targets’ (Perugini and Gordon, 2017: 5). As the International Committee for the Red Cross notes, the automation of target identification is effectively death and injury based on a generalized target profile, where human life is reduced to sensor data and machine processing (International Committee of the Red Cross (ICRC, 2021): 16). And insofar as the military apparatus of distinction is accountable to international law, its fundamental point of reference is the binary civilian/combatant. IHL has difficulty recognizing positions outside of that binary, including the possibility that one might be both or neither (Wilke, 2014). Even among those identified as combatants, IHL further requires recognition of those who are at a given moment ‘out of combat’, for example a combatant who is indicating the desire to surrender, or civilians who are not, or are no longer, taking a direct part in hostilities. Perugini and Gordon (2017) take US drone attacks in Pakistan and Israel’s 2014 attacks on Gaza as cases in point to illustrate how the apparatus of distinction constructs and frames that which is perceptible, effaces the category ‘civilian’ as it reconfigures civilian spaces as battlespaces, and becomes one of the key instruments in legitimizing an extra-legal justification for the state’s exercise of violence (2017: 9). In an argument aligned with Butler (2010), they conclude that, rather than a necessary response to the uncertainty of identification in the contemporary battlespace, the apparatus of distinction generates and relies upon its attendant ambiguities:

The increasing technological investments and epistemic operations devoted to the identification and constitution of liminal figures within theatres of war alongside the extensive legal and military analysis devoted to such figures point to the fact that liminality is rapidly being produced as part of the norm. (Perugini and Gordon, 2017: 18)

As the category of militant is expanded, the space for being counted as a civilian shrinks (see Wilke, 2014, 2017). This was made scandalously clear in the 2012 revelation in *The New York Times* that then US President Barack Obama had ‘embraced a disputed method for counting civilian casualties’, which, according to several administration officials, ‘in effect counts all military-age males in a strike zone as combatants … unless there is explicit intelligence posthumously proving them innocent’ (Becker and Shane, 2012). Moreover, the companion to an expanded category of militancy is an extended temporality of imminent threat. The Justice Department memo reported by Becker and Shane reveals that for a threat to be deemed imminent does not require the United States to have clear evidence that a specific attack on US persons and interests will take place in the immediate future. In the case of the US targeted assassination campaign in Pakistan, Ahmad (2016) notes that most of the ‘militants’ killed there are guilty by association and are neither in the process of acting aggressively nor about to do so. Conversely, he observes, there are numerous examples of known targets declared multiple times to be dead (p. 11).^15^

Idrees Ahmad’s work begins to clarify how military aspirations to perfect intelligence are reliant on ongoing technologies for the suppression of inconvenient truths. In a series of articles written for *Al Jazeera*, he casts doubt on official statistics regarding the effects of the US operations in Pakistan (Ahmad, 2011). Writing at the height of those operations, Idrees Ahmad elaborates what he names the ‘magical realism’ of their body counts, translating the genre of magical realism to ‘the production of drone statistics where the apparent rigor of method obscures a fantastical underlying reality’ (Ahmad, 2016: 4). He observes that absent any systematic process for verifying the identity of those killed, ‘the Obama administration and its Pakistani counterparts have gone out of their way to obscure realities on the ground’ (2015: 12). They are aided in this by media reports that rely on ‘official sources’ (almost always unnamed), which consistently under-report non-combatant deaths and over-estimate the numbers of ‘militants’ killed in a given operation. These numbers, he explains, are then aggregated by certain prominent ‘think tanks’ (rendering them more authoritative) and circulated further through the media.^16^ The numbers are consequently constructed through what Ahmad (2016: 3) characterizes as ‘rituals of objectivity that mask credulity’, fetishizing statistics without questioning the integrity of the data from which they are compiled. This circuit of reporting effectively contributes, he argues, to serious under-representation of the death wrought by these operations, and to ‘a misleading perception of the war as precise, surgical and discriminating’ (p. 3; see also Suchman, 2020). As long as this form of agnotology succeeds:

The myth of precision and the absence of risk make it immensely attractive for politicians to seek military solutions to political problems. Citizens are distanced from the wars fought in their names, and leaders are encouraged to use force where they otherwise might exercise caution. (Ahmad, 2016: 3)

To differentiate signal and noise, filmmaker Steyerl (2019) observes, means not only to recognize patterns but also to create them in the first place (p. 3).^17^ If the dream of omniscience erases the realities of un/intelligibility that inform actual military actions, one way of restoring those realities is through close analysis of the forensic evidence from specific incidents. A troubling case in point is provided by Wilke’s detailed account of a NATO airstrike in Afghanistan in 2009, against a large gathering of people drawn together around two trucks carrying fuel for NATO troops, which had been stolen by Taliban fighters and then become stuck on a sandbank near Kunduz (Wilke, 2017). Based on transcripts and documentary evidence assembled by the International Security Assistance Force (ISAF), Wilke shows how mandates issued by ISAF commander General Stanley McChrystal aimed at limiting use of air strikes and prioritizing the protection of the civilian population came into conflict with the readings of the scene by Provincial Reconstruction Team Commander Colonel Georg Klein and the German Joint Terminal Attack Controller (JTAC) on the ground. Those readings ultimately outranked the questions and doubts voiced by the F-15E pilots ordered in the end to drop their bombs.

Wilke explains that as the ranking German officer in charge of operations at the time, Klein was initially reliant for visual access to the situation on the grainy live video feeds from a B1 bomber high above the site. When the B1 was forced to return to base to refuel, Klein translated what was by many accounts a highly uncertain situation into the only circumstance that would warrant additional air support; that is, the assertion of an ‘imminent threat’ in the context of a ‘troops in contact’ situation on the ground. This was despite the absence of any troops on the ground, or of any clear evidence of a threat.^18^ Notwithstanding repeated questioning from the pilots, evident in the transcripts, the JTAC commander on the ground insisted to the pilots that ‘we’ve got the intel information that everybody down there is hostile’ (quoted in Wilke, 2017: 19). Klein subsequently called a strike that resulted in the deaths of over 100 people, never definitively identified by outside investigators but the great majority of whom would arguably be classified as civilians under the terms of International Humanitarian Law.

As Wilke (2017) points out, in situations like that in Kunduz, ‘the distinction between civilians and combatants is largely a visual exercise’ (p. 12). At the same time, ‘the interpretations of the visual images and the decisions on targeting are shaped by background assumptions and situated knowledges, including fantasies of race, risk, and violence’ (p. 25). Importantly for the present argument, Wilke directs our attention away from the quality of the images from the F-15E infrared cameras (problematic as that was), to the question of how those images were translated into ‘actionable intelligence’. Building on a growing literature that analyzes the scopic regimes of military visuality (Chamayou, 2014; Gregory, 2011), Wilke demonstrates how intelligence, particularly with respect to the categories of civilian/combatant, is irremediably situated in the political commitments, knowledge systems, affective registers, and temporal contingencies of military operations.

On the ground in Kunduz, those who spent time there observed, loyalties and local alliances shifted frequently (Wilke, 2017: 14). These uncertainties were not generally reported up the military chain of command, however. Instead, written reports ‘hewed to the official terminology and liberally ascribed attacks to enemies such as “irregular forces” or “Islamic networks”, “insurgents” or “Taliban” that were plausible according to the official knowledges’ (Wilke, 2017: 14). Meanwhile, as Wilke observes, the force of the bomb dropped in Kunduz obliterates not only the opportunity for identification by those in command and control, but for grief and ceremonial goodbyes by those who need no investigation to tell them whose lives have been lost. These are troubles that cannot be resolved, but only reinforced, by data analytics, reliant as they are on received categorizations.

## Expanding situational awareness as a project of demilitarization

I have focused here on the concept of situational awareness as it is mobilized and operationalized within military imaginaries. Military discourses of situational awareness presume a world existing outside of the apparatus through which that world is sensed. The challenge is to detect relevant events happening ‘out there’ in the form of signals that can be transmitted rapidly to relevant actors, in order to inform their actions. However much those actions might then affect subsequent events, the circuit of observation and action relies upon an onto-epistemology that positions war fighters as always engaged in objectively necessary interventions into a world separate from and threatening to their own.

Close analyses of actual incidents make clear, however, that the problem of military ‘intelligence’ is not simply a matter of adequate sensor networks, or access to information, or even its distribution. As all understanding is inescapably partial and situated, it is – particularly in the context of military operations – profoundly shaped by longstanding cultural historical predispositions and investments, animated by the exigencies of the moment. Crucially, rather than further incorporation into the closed-world imaginary of cybernetics, these operations need re-reading through a performative understanding of response-able perception and action (e.g. Suchman, 2016b). Read in that way, the radical openness of ‘actual reality’ poses an enduring problem for the project of AI.

This analysis suggests the need for different but related sites of situational awareness, in the form of wider public understanding of the modes of ignorance on which US militarism depends. Both investigative journalism and critical scholarship have a crucial role to play here, given the significance of their stories in shaping how relevant publics understand the conditions and effects of operations undertaken in their names. This wider frame of awareness has material consequence insofar as it affects the degrees of engagement and concern that can be mobilized either in support of or in resistance to the continuation of dominant military discourses and the practices that they legitimize. Expanding situational awareness would require an inversion of current practices so that all of those killed in an operation would be assumed innocent until an administration was able to prove otherwise. Soon after the publication of Idrees Ahmad’s critique, the Bureau of Investigative Journalism’s drones project was launched and has now evolved into the most comprehensive and reliable database of information on the war’s effects. The Bureau continues to follow stories of incidents as they develop; it works with journalists, researchers and lawyers representing the affected persons to develop a more rigorous accounting of those being killed.^19^ This is the only form of knowledge production, Idrees Ahmad argues, that can take us beyond magical realism.

On this understanding situational awareness in the context of counterterrorism is a technology specifically aimed at those who live in what are extraordinarily secure spaces, so that it takes these ongoing interventions to generate the level of public anxiety required to support militarism as the pre-eminent form of US foreign policy. We might contrast the situational awareness promoted by the US national security state to those growing areas of the world where existential danger is far from inchoate, but instead is an affective and corporeal reality of everyday life. As Somali-British poet Warsan Shire poignantly remarks of the current crisis of refuges in the Mediterranean, no one puts their children in a boat unless the water is safer than the land (Shire, 2013).

## Conclusion

The figure of artificial intelligence, while proffered as a technological solution to the irremediable contingencies of war fighting, is rather the latest investment in a project of containment on the part of the United States military establishment. The aspiration to closure, integral to the logics of an international order based in military dominance, propels the destructiveness of a US foreign policy that regenerates the insecurities that it ostensibly eradicates. The closed world relies, moreover, on forms of systemic ignorance required to maintain the premise that war fighting can be conducted rationally through a seamless web of technologically generated situational awareness.^20^

The through line from the closed world of the Cold War to the current technopolitical imaginary is the figure of objective knowledge that can resolve interpretive flexibility, this time through data analytics at scale. The premise is that the analytic apparatus can transform the contingencies and ambiguities of relations on the ground into noise from which, through statistical techniques operating over large data sets, a stable and unambiguous signal can be abstracted. This premise rests upon the conflation of signals with information, through erasure of the situated knowledges through which information is produced. The technopolitical imaginary of containment is further sustained by an unreconstructed objectivist conception of situational awareness, modeled as a cognitive system (exemplified by the OODA loop), which renders the openness of sites of operation, including military actors’ own effects on those sites, either as ‘input’ to the system or as outside the system’s boundaries. In the current moment increasing datafication, as the infrastructure for situational awareness, produces new needs for ever more complex connectivity and analytical tools, which in turn exacerbate the very complexity that they aspire to tame.^21^

I have suggested that the most powerful alternative to closed-world knowledge making is investigative journalism and other modes of on-the-ground research and reporting.^22^ These accounts convey the radical openness of war, foregrounding its associated injuries, challenging the military’s attempt to make clean demarcations where there are none to be made, and demonstrating knowledge-producing practices that do not fit the military’s imaginaries of omniscience. The ‘fog of war’ on this analysis takes on a different aspect, not as a naturally occurring atmospheric condition, or even the effect of deliberate obfuscation directed by one set of combatants at another, but as the routine manipulation of information by relevant governments to effectively keep their publics in the dark, and in that way to control the situational awareness of those in whose names the conflict is fought. At the same time, the irremediable uncertainty of war fighting, in its refusal to be contained, holds open the spaces of resistance to closed-world logics and makes evident the urgent need for demilitarization and reinvestment in more just pathways to our collective security.

## Notes

1. The Revolution in Military Affairs (RMA) is a recurring trope in military theory, marking what is taken to be an historical shift in the conduct of war requiring requisite transformations in strategy, technologies, and organization. Attributed within US military doctrine to Andrew Marshall, named by Defense Secretary James Schlesinger as the first DoD Director of Net Assessment in the early 1970s, the RMA doctrine developed in the context of the Cold War subsequently morphed in the 1990s into aspirations for algorithmically enabled, networked integration of forces examined here. The ‘fog of war’, a metaphor introduced by von Clausewitz (1984) in his classic military treatise *On War (1832)*, is that which all military intelligence aspires to dispel. The phrase became more widely known through the film *The Fog of War: Eleven Lessons from the Life of Robert S McNamara* (Morris, 2004)in which the former US Secretary of Defense reflects on the experiences of Vietnam. As Shapiro (2005) argues, critical engagements like Morris’ film shift the locus of the fog of war from the battlefield to the figures of the enemy that are generated within military discourses and institutions, a point that is central to the argument here (see also Cronin, 2008; Davidson, 2011; Owens, 2000).

2. The ‘imaginary’ is a generative trope that references ‘collectively held, institutionally stabilized, and publicly performed visions of desirable futures, animated by shared understandings of forms of social life and social order attainable through, and supportive of, advances in science and technology’ (Jasanoff and Kim, 2015: 5; see also Hecht, 2011; McNeil et al., 2017).

3. I treat the term ‘AI’ as a figure to emphasize that more than a specific technology, or even set of technologies, AI has become what anthropologist Lévi-Strauss (1987) first called a ‘floating signifier’, a term that suggests a concrete referent, but works to ‘float’ or even to evade definition in order to maximize its suggestive power. That qualification should be read into my use of the term throughout this article.

4. As the war on terror has proven intractable, we see a return to ‘superpower’ doctrine with respect to Russia and China. While a full exploration of that shift falls outside the bounds of this article, the renewed arms race that results centers AI and serves as a key referent in the promotional efforts of the contemporary military-commercial-academic complex. As the closed-world imaginary was forged in the crucible of the Cold War, there is every reason to anticipate its further revival in the insistence on an AI arms race and ‘great power’ conflict as defining the current moment.

5. In a press briefing held on March 18, 2022, to announce the latest iteration of the Joint All Domain Command and Control (JADC2) strategic plan, Lieutenant General Dennis Crall, Director, Command, Control, Communications and Computers/Cyber, cites Boyd’s OODA loop calling it ‘as relevant as ever’. See https://www.defense.gov/News/Transcripts/Transcript/Article/2972552/marine-lt-gen-dennis-crall-briefs-media-on-joint-all-domain-command-and-control/ (accessed 22 April, 2022). The JADC2 is discussed below.

6. We might ask when it was not the case that US forces operate at a distance from home. The US has approximately 800 bases around the world at the time of this writing.

7. Initially conceived by Lowell Wood of the Livermore Lab in 1986, the Brilliant Pebbles concept was aggressively marketed to the Reagan administration by Wood and his mentor Edward Teller as part of the Strategic Defense or ‘Star Wars’ Initiative. Brilliant Pebbles were imagined as artificial ‘rocks’ that would be launched into low orbit, packed full of micro-electronics, high-tech sensors, and thrusters. These rocks would lie in wait in orbit, conserving energy, until their sensors detected the launch of a Soviet ICBM. They would then spring into action automatically and autonomously, activating their thrusters to maneuver themselves into the path of enemy missiles to destroy them, throw them off course, or prevent them from dispersing their nuclear warheads. For a defense of the concept see Rowny (1990).

8. Marcuse served in this position from August 2016 to March 2020. In May of 2020 took up a position as head of strategy and innovation for the global public sector at Google. He serves on the Board of Directors of the Defense Entrepreneurs Forum (https://medium.com/defense-entrepreneurs-forum/defs-board-of-directors-joshua-marcuse-4e4105846154, accessed 25 May, 2021).

9. In January of 2019 I received an invitation from Aaron Schumacher, Research Director for the DIB, to participate in an AI Ethics roundtable at Stanford University. While I participated in the event on April 26^th^ of that year, I found myself unable to either contribute or intervene effectively given the event’s framing, which placed all my concerns – ranging from just what ‘AI’ comprises to the un/lawfulness of current US targeted assassination practices – outside of the bounds of our mandate to deliver a set of AI ethics principles to the DoD.

10. I have not been able to determine whether the two Shanahans are related.

11. In opening the session, Chair Eric Schmidt affirmed the Commission’s commitment to ‘openness and transparency’. In fact, it required a lawsuit by the Electronic Privacy Information Center (EPIC) under the Freedom of Information Act (FOIA) and Federal Advisory Committee Act (FACA) to force the Commission’s transparency obligations (see https://epic.org/foia/epic-v-ai-commission/, accessed June 2021).

12. The questions raised here go beyond the scope of this article but see Suchman (2016a).

13. Rob Carey, former Navy CIO and deputy DoD CIO, now president of Cloudera Government Solutions, states the promise this way: ‘There are tools that enable real-time decision-making – informed decision-making – at the speed of warfare. What is the basis of that? It’s cloud. It is having your data connected and in a place where I can run analytics on it in real time or near real time, and then render informed decisions to support war-fighting missions’ (Eversden, 2021).

14. For a close analysis of the self-referential nature of data ontologies see Jaton (2017), and for a broader examination of the ways in which algorithmic systems reproduce discriminatory histories see Noble (2018).

15. In the case of Pakistan between 2004 and 2015, analyses by the Bureau of Investigative Journalism show that fewer than 4% of those killed were positively identified as members of Al-Qaeda and only an additional 2% as other individuals designated as ‘high value targets’. In the end, as Ahmad (2015) points out, the identity of the majority of the dead remain unknown to those who kill them. Meanwhile, those positively identified as so-called high value targets experience multiple rounds of death and resurrection, e.g. ‘Baitullah Mehsud, the former commander [of the Taliban in Pakistan], was reported dead in drone strikes on 16 different occasions before his actual death on 5 August 2009’ (p. 11). (See also Airwars https://airwars.org/civilian-casualties, accessed 15 May, 2021.)

16. Idrees Ahmad names the Long War and New America Foundation as the primary organizations involved in this practice.

17. On the generative properties of machinic pattern recognition, Steyerl cites the example of Google’s ‘inceptionism’ or ‘deep dreaming’, produced through the application of image recognition filters to random noise, which results in the generation of shapes recognizable to us as the phantoms of images from previous training data (most notably, eyes): ‘The point is that in order to “recognize” anything, neural networks need to be taught what to recognize. Then, in a quite predictable loop they end up “recognizing” the things they were taught. … They reveal how signal and noise are defined by preexisting categories and probability’ (pp. 8–9).

18. As Wilke explains, this call was made in the context of growing hostility on the part of the local Afghan population toward NATO operations in the area, including the deaths in a suicide attack, in June of that year, of two German soldiers based in Kunduz.

19. Even then, the Bureau qualifies its estimates as conservative, counting as civilians only those who can be verified as unaffiliated with militant groups (Ahmad, 2016: 12).

20. A growing body of scholarship takes up the power of ignorance (e.g. Proctor and Scheibinger, 2008).

21. This turn to data and technosolutionism more broadly is accompanied by the dismantling of institutions for alternative forms of conflict resolution. A closely associated project to the expanded situational awareness called for here is the restoration of grounded diplomacy (see Farrow, 2018).

22. An exemplary case of the latter appeared during the completion of this paper, in the form of Azmat Khan’s Civilian Casualty Files (2021), released as an interactive series in the *New York Times*. The series won the 2022 Pulitzer Prize for International Reporting.
